# Subunit-Specific Role of NF-κB in Cancer

**DOI:** 10.3390/biomedicines6020044

**Published:** 2018-04-17

**Authors:** Barbara Kaltschmidt, Johannes F. W. Greiner, Hussamadin M. Kadhim, Christian Kaltschmidt

**Affiliations:** 1 AG Molecular Neurobiology, University of Bielefeld, 33615 Bielefeld, Germany; Barbara.Kaltschmidt@uni-bielefeld.de; 2 Department of Cell Biology, University of Bielefeld, 33615 Bielefeld, Germany; johannes.greiner@uni-bielefeld.de (J.F.W.G.); kadhim.hussamadin@uni-bielefeld.de (H.M.K.)

**Keywords:** NF-κB, RELA, cREL, RELB, tumor, cancer, transformation, inflammation, gene expression, tumor necrosis factor, Treg

## Abstract

The transcription factor NF-κB is a key player in inflammation, cancer development, and progression. NF-κB stimulates cell proliferation, prevents apoptosis, and could promote tumor angiogenesis as well as metastasis. Extending the commonly accepted role of NF-κB in cancer formation and progression, different NF-κB subunits have been shown to be active and of particular importance in distinct types of cancer. Here, we summarize overexpression data of the NF-κB subunits RELA, RELB, and c-REL (referring to the v-REL, which is the oncogene of Reticuloendotheliosis virus strain T) as well as of their upstream kinase inhibitor, namely inhibitor of κB kinases (IKK), in different human cancers, assessed by database mining. These data argue against a universal mechanism of cancer-mediated activation of NF-κB, and suggest a much more elaborated mode of NF-κB regulation, indicating a tumor type-specific upregulation of the NF-κB subunits. We further discuss recent findings showing the diverse roles of NF-κB signaling in cancer development and metastasis in a subunit-specific manner, emphasizing their specific transcriptional activity and the role of autoregulation. While non-canonical NF-κB RELB signaling is described to be mostly present in hematological cancers, solid cancers reveal constitutive canonical NF-κB RELA or c-REL activity. Providing a linkage to cancer therapy, we discuss the recently described pivotal role of NF-κB c-REL in regulating cancer-targeting immune responses. In addition, current strategies and ongoing clinical trials are summarized, which utilize genome editing or drugs to inhibit the NF-κB subunits for cancer treatment.

## 1. The NF-κB Family—An Introduction

The transcription factor nuclear factor “kappa-light-chain-enhancer” of activated B-cells (NF-κB) [1,2] plays a key role in a broad range of cellular processes like cell growth, apoptosis, inflammation, learning, and memory as well as immunity [3,4]. The transcription factor is ubiquitously expressed and responds to diverse stimuli, particularly including infectious agents, cytokines, or growth factors [5,6]. According to its various cellular functions, deregulation of NF-κB signaling is strongly associated with cancer formation and progression [7,8].

**Figure 1:**
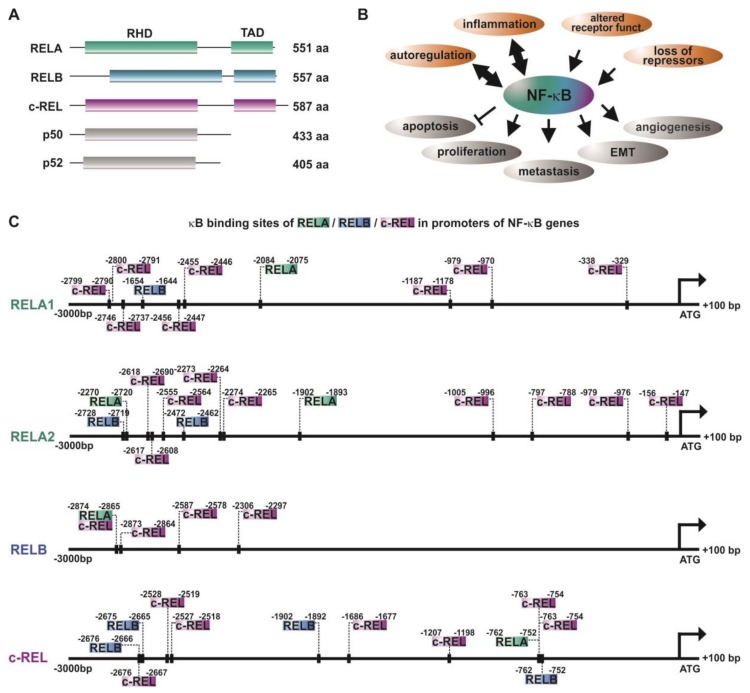
NF-κB and autoregulation of NF-κB subunits in cancer. (**A**) Schematic view of the NF-κB-family ([11]). (**B**) Principal mechanisms causing overexpression/activation of NF-κB as well as the cellular effects of NF-κB acitivity leading to cancer development and progression. RHD: REL homology domain, TAD: transactivation domain. (**C**) The promoters of NF-κB subunits RELA, RELB, and c-REL contain various κB sites enabling autoregulation of NF-κB in cancer. Promoter analysis was done as described in [12]. Briefly, sequences of promoter regions (3000 bp downstream and 100 bp upstream of the respective ATG) of interest were taken from Eukaryotic Promoter Database (epd.vital-ti.ch) for Homo sapiens. The binding sites for the gene of interest in the chosen promoter sequence were looked up using the JASPAR Tool [13]) with a relative score threshold of 85%.

The NF-κB family is composed of five subunits, namely, RELA (p65), RELB, c-REL, p50, and p52 (Figure 1A), all comprising a conserved REL homology domain (RHD) near the N-terminus. This domain is crucial for DNA binding (N-terminal part of RHD), dimerization of NF-κB family members, as well as interaction with the inhibitors of κB (IκBs) (C-terminal part of RHD). Via the RHD, NF-κB family members can form homo- or heterodimers, like p50/RELAp65, RELB/p50, p52/c-REL, or RELA/RELA. In addition, the subunits RELA, RELB, and c-REL contain a C-terminal transactivation domain (TAD) [9,10].

Inactive NF-κB dimers are localized within the cytoplasm, since the NLS (nuclear localization signal) within the RHD is masked by IκBs. During canonical NF-κB signaling, binding of ligands such as cytokines, growth factors, or lipopolysaccharides to their respective receptors (see below, Section 2) leads to the phosphorylation of the IκB kinase (IKK) complex comprised of IKK1/IKK2 (IKKα/IKKβ) and NEMO (NF-κB essential modulator). Phosphorylated IKKs, in particular IKK2, in turn phosphorylate IκBα, which subsequently undergoes proteasome-mediated degradation via polyubiquitinylation. Degradation of IκBα leads to demasking of the nuclear translocation site of the NF-κB p50/RELA heterodimer. In turn, translocation into the nucleus occurs. This results in the expression of NF-κB-target genes via binding to the respective target sites [4,9]. On the contrary, non-canonical NF-κB signaling induced by distinct members of the tumor necrosis factor (TNF) family like lymphotoxin-β relies on the phosphorylation of IKK1 via NIK (NF-κB-inducing kinase). IKK1 mediates the phosphorylation of p100, associated to RELB, inducing the proteasomal processing of p100 to p52 [14]. The p52/RELB heterodimer is able to enter the nucleus and activate specific target genes via binding to selective κB sites. Both the canonical and the non-canonical pathway have been described to be closely linked to cancer formation and progression [15] (Figure 1B, see also Section 2). In addition, atypical NF-κB pathways, as in the case of epidermal growth factor receptor (EGFR) tyrosine kinase-dependent NF-κB activation, were likewise described to promote cancer [16]. 

## 2. NF-κB in Inflammation and Cancer

In response to physical or physiological stress, injury, or infection, inflammation takes place as a key defense process of innate immunity aiming to restore the physiological situation. NF-κB is broadly described to be one of the key transcription factors regarding pro-inflammatory signaling, particularly activated by the presence of pro-inflammatory cytokines (like TNFα or IL-1), lipopolysaccharides (LPS) of the bacterial cell wall [17], or viral and bacterial nucleic acids [18]. Recognition of cytokines or LPS species is mediated by the respective receptors, such as TNF receptors or Toll-like microbial pattern recognition receptors (TLRs). As described above, binding of such ligands to their respective receptors leads to canonical NF-κB signaling, ultimately resulting in the translocation of released NF-κB p50/RELA into the nucleus and binding onto κB elements located in distinct target genes. Among the broad range of target genes of NF-κB, the most prominent ones in terms of inflammation are also pro-inflammatory cytokines, such as TNFα [19,20], IL-1 [21], and T cell regulatory ones, such as IL-2 [22] (proliferation) or IL-8 [23] (recruitment). The resulting feed-forward loops of NF-κB-activation, particularly in the case of TNFα, make NF-κB a booster of pro-inflammatory signaling, which augments the inflammation. In the case of cancer, these signaling cascades and the resulting production of pro-inflammatory cytokines likewise recruit cytotoxic immune cells targeting and eliminating the transformed cells [24]. However, the presence of active NF-κB in cancer is a double-edged sword. Although being a mediator of immune responses eliminating cancer cells, NF-κB was observed to be constitutively active in many types of cancer arising from a prolonged chronic inflammatory microenvironment or induced by various oncogenic mutations [8,25]. In a seminal review, Baud and Karin listed 11 types of blood-born cancers (including frequent ones such as acute myeloid leukemia (AML)) and 23 solid tumors (including frequent ones such colon cancer), which showed activated NF-κB signaling [26]. By way of example, elevated NF-κB activity resulting in the accumulation of pro-inflammatory cytokines in the tumor was reported to directly contribute to a pro-tumorigenic microenvironment in colon cancer [27]. Despite this close relation between inflammatory NF-κB signaling and cancer, NF-κB directly mediates vital tumor-promoting mechanisms. NF-κB activity was shown to stimulate cell proliferation, prevent apoptosis, and promote tumor angiogenesis, epithelial-to-mesenchymal transition, invasiveness, as well as metastasis [8,28,29] (Figure 1B). For further details, see a recent review by Taniguchi and Karin [30]. Extending this commonly accepted role of NF-κB in cancer formation and progression, different NF-κB subunits have been shown to be active and of particular importance in distinct types of cancers [11]. In the following, we will discuss the current literature depicting the roles of different NF-κB subunits, their autoregulation, and specific transcriptional activity in cancer and outline how particular subunits and upstream kinases contribute to cancer progression.

## 3. Autoregulation of NF-κB—A Potential Driver on the Road to Cancer Development?

In addition to the canonical, non-canonical, and atypical activation of NF-κB (see also Section 1), NF-κB RELA, RELB, and c-REL have been described to be activated by autoregulation [31,32,33,34]. Accordingly, the promoter analysis of NF-κB-subunits performed in the present study depicted the presence of various κB-binding sites for RELA, RELB, and c-REL (Figure 1C). While RELA and c-REL promoters contain binding sites for all three transactivating subunits RELA, RELB, and c-REL, the promoter of RELB showed only binding sites for cREL and RELA/c-REL in its proximal region. Next to the transactivating subunits, p50 and p52 are likewise known to be autoregulated [35,36]. As depicted in Table 1, several tumor types show various levels of overexpression of the NF-κB-transactivating subunits. A mechanistic reason for this observation might be a feed-forward autoregulation. In this line, a broad range of different κB binding sites within the NF-κB promoters shown here suggest NF-κB feed-forward loops to act as boosters of vital tumor-promoting mechanisms, like cell proliferation, angiogenesis, invasiveness, and metastasis. In addition, these autoregulatory mechanisms may at least in part account for the constitutive activity of NF-κB observed in a broad range of cancers [25,26].

## 4. Activity of Distinct NF-κB Upstream Kinases in Cancer

To investigate the role of the upstream regulators of NF-κB-signaling IKK1 and IKK2 in human cancers, we applied database mining using COSMIC to determine their levels of overexpression (Table 2) [37,38].

**Figure 2:**
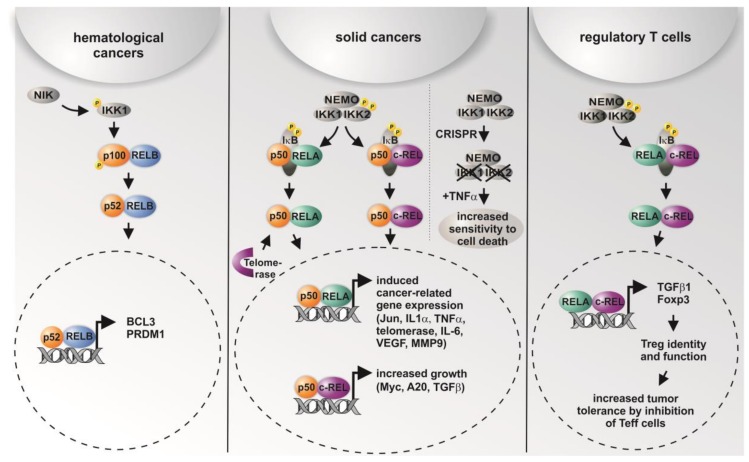
Graphical overview on the differential roles of NF-κB subunits and their transcriptional activity in distinct types of cancer and in regulatory T cells. While non-canonical signaling is mostly present in hematological cancer, solid cancer shows predominantly canonical signaling via p50/RELA or p50/cREL. In addition, CRISPR/Cas-mediated double knockout (KO) of IKK1/2 was recently shown to result in increased sensitity to TNF-α-mediated cell death [38]. In regulatory T cells (Tregs), activation of RELA/cREL results in distinct target gene expression leading to active Tregs inhibiting effector T cells (Teff), which infiltrate the tumor [11,12,52,55,56,57,58].

Here, IKK1 and IKK2 showed distinct levels of overexpression in different types of cancer, with IKK2 being overexpressed in cancers arising in the large intestine, the oesophagus, and the lung (Table 2). Accordingly, data from a lung cancer mouse model indicated that tumor cell proliferation was significantly impaired after deletion of IKK2 [39]. Interestingly, IKK-mediated phosphorylation of IκB was shown to mainly depend on the IKK2 catalytic subunit of the IKK complex in mice [40], particularly in terms of prevention of apoptosis [41]. On the contrary, we recently observed TNF-α-mediated cell death only in human cells lacking IKK1 and IKK2 and not in single CRISPR/Cas-mediated IKK knockouts, suggesting that both IKK1 and IKK2 are required for functional TNF-signaling [38] (Figure 2). However, knockout of IKK2 was shown to be associated with about a one-third reduced number of tumors in a colitis-associated cancer model. Surprisingly, deletion of IKK2 in enterocytes led to an increased expression of COX-2, IL-6, and MIP-2, whereas TNF-α, IL-1, and ICAM were not affected. In the myeloid compartment, the number of tumors per mouse was reduced by about 50% after deletion of IKK2 [42]. Constitutive IKK2 activation in intestinal epithelial cells was further demonstrated to induce intestinal tumors in mice [43]. These findings are in accordance with the profound overexpression of IKK2 observed in cancers of the large intestine (Table 2) [38]. On the functional level, IKK2 was shown to directly promote the development of lung cancer in an inflammation-dependent manner triggered by tobacco smoke, which was abrogated by ablation of IKK2 in myeloid cells [44]. Applying a model of breast cancer progression, Huber and colleagues showed IKK2-dependent activation of NF-κB to be essential for epithelial-to-mesenchymal transition and metastasis [45]. Furthermore, the activation of NF-κB by overexpression of constitutively active IKK-2 in prostate cancer cell lines promoted the growth of prostate cancer cells in bone [46]. Accordingly, IKK1 activated by cytokines was shown to control prostate cancer metastasis, with the amount of active nuclear IKK1 correlating with metastatic progression of mouse and human prostate cancer [47].

Next to IKKs, downstream signaling of IκBs is likewise associated to cancer development and progression. Pikarsky and colleagues reported a super-repressor of IκB in hepatocytes to act as a tumor promoter in inflammation-induced liver cancer [48]. Furthermore, in Hodgkin’s disease, a hematologic malignancy, the overexpression of a truncated form of IκB is linked to constitutive NF-κB (p50/RELA) activity [49]. In addition, we observed reduced cell growth and a retarded G1/S transition in human cercival cancer cells, accompanied by an increase in cyclin D1-dependent kinase activity after overexpression of IκBα. We further demonstrated a crosstalk of IκBα overexpression with cell cycle checkpoints via a reduction of transcription factor p53 and elevation of p21WAF [50].

## 5. Differential Roles of NF-κB Subunits in Cancer

To provide an overview on the occurrences of distinct NF-κB subunits in cancer subtypes, we assessed the overexpression of the NF-κB subunits RELA, RELB, and c-REL in human cancers by database mining, using COSMIC (Table 1) [12,37]. 

In line with the concept of subunit-specific gene regulation in cancer [11], we found profound differences in the overexpression of particular NF-κB subunits in distinct types of cancer. On the contrary, gene amplification and/or mutations within the coding region were only found in neglectable amounts in the COSMIC database. For instance, RELA is most dominantly overexpressed in ovarian cancer and cancer of adrenal glands in comparison to RELB and c-REL, while the overexpression of c-REL is most abundantly found in lung cancers, compared to that of the other subunits (Table 1). In 2016, Scheidereit and coworkers reported the cell survival of Hodgkin lymphoma (HL) cells to be predominantly controlled by the non-canonical NF-κB pathway. In particular, knockdown of p52/RELB in HL cells resulted in 95% reduction of viability. Using combined ChIP-sequencing and microarray analyses, the authors further showed a low frequency of RELA bound to DNA, but a high frequency of DNA-bound p50- and p52-containing complexes, also including p50/p52 heterodimers [51]. Non-canonical NF-κB signaling was further reported to be active in 20% of the samples from 155 multiple myeloma patients. Here, constitutive activation of the non-canonical RELB/p52 pathway was associated with abnormalities like bi-allelic deletion events, mutations, and gene rearrangements in the genes *NFKB1 (p50/p105)* and *NFKB2* (*p52/p100*) [52]. Furthermore ectopic expression of RELB can inhibit the growth of tumor xenografts in mice [53]. C-REL is frequently amplified in B cell lymphoma and could function as a tumor-promoting transcription factor, but c-rel-/-mice also could develop an earlier onset of B cell lymphoma [54]. In summary, non-canonical NF-κB-signaling seems to predominantly contribute to hematological cancers (Figure 2). 

In contrast to its non-canonical counterpart, canonical NF-κB signaling is described to be present in solid cancers (Figure 2). Shukla and colleagues reported an increased expression of RELA and p50 in human high-grade prostate adenocarcinomas, leading to constitutive NF-κB activity. Active NF-κB p50/RELA led to increased expression of NF-κB target genes *MMP9* and *VEGF*, commonly involved in cell migration and vascularization. Accordingly, NF-κB activity was related to tumor progression due to transcriptional regulation of these NF-κB target genes [57,59]. An increased NF-κB RELA signaling was likewise observed in tumor-initiating stem-like cells in human prostate cancer [60]. Applying a set of 1826 fully annotated prostate cancers, Gannon and colleagues showed a significant association between an increase in the nuclear frequency of NF-κB RELA and Gleason score, which is used to score prostate cancer grade, although the contribution of NF-κB RELA to a post-surgical predictive model appears modest [61]. In lung cancer, NF-κB RELA is known to be required for K-Ras-induced lung tumorigenesis, while lung tumors with RELA deletion show increased apoptosis accompanied by reduced spread and a lower grade [62]. In addition, Mukhopadhyay and colleagues showed highly increased levels of NF-κB p50 in nine of 11 non-small-cell lung carcinoma tissues [63]. KrasG12D-induced IKK2/NF-κB activation, resulting in increased expression of IL1-α and p62 and respective feed-forward loops, was demonstrated to be required for the development of pancreatic ductal adenocarcinoma [64]. In 2003, Nair and coworkers showed a constitutive activation of NF-κB RELA during human cervical cancer progression. Here, NF-κB RELA was demonstrated to be particularly activated in high-grade squamous intraepithelial lesions and squamous cell carcinomas of the human uterine cervix [65]. Interestingly, NF-κB-dependent transcription was recently shown to be directly regulated by telomerase. In particular, telomerase directly bound to the NF-κB RELA subunit, thus regulating NF-κB-dependent gene expression by binding κB sites in the promoter regions of IL-6 and TNF-α, both critical for inflammation and cancer progression [58] (Figure 2). The effect of telomerase on the strong activation of colony formation of tumor stem cells could be repressed by siRNA knockdown of RELA. Given the transcriptional regulation of telomerase by NF-κB RELA, Gosh and coworkers suggested a feed-forward regulation, linking chronic inflammation to increased activity of telomerase in human cancer [58]. Next to RELA, the NF-κB subunit c-REL was likewise shown to possess a key role in tumor formation. In 2000, Cogswell and colleagues revealed the induction of mammary tumors by c-REL expression in mouse models of breast cancer [66]. Shehata and coworkers further demonstrated a sixfold slower cell growth in cultivated cervical cancer cells after expression of the c-REL homolog Xrel3 from *Xenopus laevi* [67]. We recently investigated the role of c-REL in human cervical cancer cells using CRISPR/Cas9n-mediated gene editing. Knockout of c-REL resulted in significantly decreased basal expression levels of Myc, A20, and TGFβ, accompanied by a significantly reduced proliferative behavior and a significant delay in the prometaphase of mitosis (see also Figure 2 for overview). Compared to the wild type, an increased resistance against chemotherapeutic agents was observable in c-REL knockout cells [12]. Next, by directly promoting cancer cell growth and proliferation, c-REL was very recently shown to possess an important role in cancer-targeting immune responses with highly promising implications for therapeutic approaches [68]. Enabling tumor progression, activated CD4^+^Foxp3^+^ regulatory T cells (Tregs) are known to migrate to the tumor site and inhibit of CD8 effector T cells (Teffs), which are mainly responsible for anti-tumor immune responses [69]. In melanoma, large amounts of Tregs have been observed [70] and associated with impaired prognosis, while a lesser amount of Tregs was associated with increased survival in stage 4 melanoma patients [71]. In their groundbreaking study, Grindberg-Bleyer and colleagues demonstrated NF-κB cREL as the critical subunit for identity and function of activated CD4^+^Foxp3^+^ Tregs in melanoma (see also Figure 2). Notably, deletion or inhibition of c-REL, but not of RELA, in Tregs resulted in reduced melanoma growth and potentiated anti-PDL1 therapy, a ligand presented by cancer cells and dendritic cells to evade the immune system by binding to the immunosuppressive programmed death (PD) receptor on CD8^+^ Teff cells [68]. In the following, we will emphasize the therapeutic implications of these findings as wells current strategies to utilize genome editing or drugs for targeted deletion/inhibition of NF-κB subunits in cancer therapy.

## 6. Targeting NF-κB Subunits via Genome Editing or Drugs—Therapeutic Implications

Given the important roles of distinct NF-κB subunits in cancer development and progression, we aim to summarize currently used drugs targeting NF-κB subunits for cancer treatment. One drug utilized in the clinics is the NF-κB inhibitor Bortezomib [72] (developed by Millenium Pharmaceuticals (Cambridge, MA, USA) as Velcade, also known as Neomib (Getwell Pharmaceuticals, Gurgaon, Haryana, India) or Bortecad (Cadila Healthcare, Ahmedabad, Gujarat, India), a reversible 26S proteasome inhibitor of IκB-α degradation. This drug is certified in Europe as monotherapy for pre-treated adult patients with progressive multiple myeloma. Next to Bortezomib, the NF-κB inhibitor Thalidomide is also clinically applied. In 2002, Majumdar and colleagues showed Thalidomide to abrogate TNFα-dependent activation of IKKs and I-Bα [73]. First evaluated in patients with refractory multiple myeloma in the 1990s, Thalidomide is now known to cause responses in 30–50% of myeloma patients as a single agent and acts synergistically with corticosteroids and chemotherapy [74,75]. In addition, a phase III clinical trial is presently studying a combination of Aspirin (an IKK inhibitor) and Esomeprazole (a proton pump inhibitor) to prevent esophageal cancer in patients with Barrett’s metaplasia (ClinicalTrials.gov Identifier: NCT00357682). Furthermore, a phase 3 clinical trial is using high-dose ascorbic acid, a well-known NF-κB inhibitor [76], as a pharmaceutical for a combination therapy for colorectal cancer (ClinicalTrials.gov Identifier: NCT02969681). Recently, a subunit-specific inhibitor for c-REL was discovered, which might be useful for inhibiting Tregs (patent filed, IPO: WO2017058881A1). Thus, the inhibition of c-REL might be a new way to treat tumors pharmacologically. In addition to the application of NF-κB-inhibiting drugs, a recent increase in clinical studies applying CRISPR/Cas-mediated knockout strategies suggest that gene therapy might be considered in future therapeutic approaches (e.g., five clinical trials with PD-1 knockout engineered T cells; information retrieved in February 2018 from ClinicalTrials.gov). 

## 7. Conclusions

Although NF-κB might be considered as a major factor in cancer development and progression, distinct NF-κB subunits seem to be active in different kinds of cancer. While non-canonical NF-κB RELB signaling is described to be mostly present in hematological cancers, solid cancers reveal canonical NF-κB RELA (p65) and/or c-REL activity. These particular subunits contribute to cancer formation and invasiveness as a result of their specific transcriptional activity, inter alia via feed-forward loops as in the case of TNFα or telomerase. Currently ongoing clinical trials target NF-κB-dependent signaling by application of drugs or CRISPR/Cas-mediated genome editing impinging on potentially NF-κB-driven processes. Thus, although the here summarized data emphasize the importance to assure subunit specificity, NF-κB seems to be a highly promising target for cancer treatment. Michael Karin and coworkers suggested over the years a universal activation of NF-κB in cancer by inflammatory cytokines [30]. It might be important to note that our analysis of the COSMIC database argues against this universal mechanism of cancer-mediated activation of NF-κB. Here, we suggest a much more elaborated mode of NF-κB regulation in terms of a tumor type-specific upregulation of the NF-κB subunits.

## Figures and Tables

**Table 1:** Overexpression of NF-κB subunits in distinct human cancer tissues. COSMIC was used for database mining [37]. Parts of this table are published in part [12]). n.a: not assessed.

Cancer Tissue	RELA		RELB		c-REL	
	% Overexpressed	No. Tested	% Overexpressed	No. Tested	% Overexpressed	No. Tested
Ovary	11.65	266	3.38	266	7.52	266
Lung	2.36	1019	4.12	1019	7.26	1019
Urinary tract	2.45	408	4.41	408	7.11	408
Endometrium	1.99	602	8.8	602	6.81	602
Pancreas	2.79	179	6.7	179	6.7	179
Haematopoietic and lymphoid	4.07	221	1.36	221	6.33	221
Soft tissue	3.42	263	1.9	263	6.08	263
Cervix	1.3	307	7.17	307	5.86	307
Upper aerodigestive tract	2.49	522	4.02	522	5.75	522
Kidney	2.83	600	4.5	600	5.5	600
Thyroid	1.36	513	3.7	513	5.46	513
Large intestine	1.87	610	5.25	610	4.92	610
Stomach	7.02	285	7.37	285	4.91	285
Liver	3.75	373	6.97	373	4.83	373
Central nervous system(CNS)	4.45	697	3.73	697	4.73	697
Prostate	4.62	498	5.02	498	4.62	498
Breast	4.17	1104	4.26	1104	3.71	1104
Skin	6.34	473	4.23	473	3.59	473
Oesophagus	2.4	125	2.4	125	3.2	125
Adrenal gland	12.66	79	5.06	79	2.53	79
Nervous system (NS)	n.a.	n.a.	n.a.	n.a.	n.a.	n.a.
Bone	n.a.	n.a.	n.a.	n.a.	n.a.	n.a.

**Table 2:** Overexpression of IκB kinases IKK1 and IKK2 in distinct human cancer tissues. COSMIC was used for database mining [37].

Cancer Tissue	IKK1		IKK2	
	% Overexpressed	No. Tested	% Overexpressed	No. Tested
Breast	7.07	1104	9.6	1104
Lung	5.1	1019	7.16	1019
Adrenal Gland	5.06	79	1.27	79
Endometrium	4.98	602	13.12	602
Oesophagus	4.8	125	24.8	125
Liver	4.56	373	5.36	373
Pancreas	4.47	179	4.47	179
Urinary tract	4.41	408	4.9	408
Stomach	4.21	285	7.72	285
Ovary	4.14	266	7.52	266
Thyroid	4.09	513	2.34	513
Prostate	3.21	498	5.02	498
Haematopoietic and lymphoid	3.17	221	5.43	221
Upper aerodigestive tract	2.87	522	6.13	522
Large intestine	2.46	610	18.52	610
Central nervous system(CNS)	2.44	697	3.59	697
Cervix	1.95	307	5.54	307
Soft tissue	1.9	263	6.08	263
Kidney	1.83	600	3.33	600
Skin	1.48	473	8.25	473
Biliary tract	n.a.	n.a.	n.a.	n.a.
Bone	n.a.	n.a.	n.a.	n.a.
Nervous system (NS)	n.a.	n.a.	n.a.	n.a.
Pituitary	n.a.	n.a.	n.a.	n.a.
Salivary gland	n.a.	n.a.	n.a.	n.a.
Testis	n.a.	n.a.	n.a.	n.a.
